# Probiotic Bacteria Alter Pattern-Recognition Receptor Expression and Cytokine Profile in a Human Macrophage Model Challenged with *Candida albicans* and Lipopolysaccharide

**DOI:** 10.3389/fmicb.2017.02280

**Published:** 2017-11-29

**Authors:** Victor H. Matsubara, Karin H. Ishikawa, Ellen S. Ando-Suguimoto, Bruno Bueno-Silva, Atlas E. M. Nakamae, Marcia P. A. Mayer

**Affiliations:** ^1^Dental School, Oral Health Centre of Western Australia, The University of Western Australia, Perth, WA, Australia; ^2^Laboratory of Oral Microbiology, Department of Microbiology, Institute of Biomedical Sciences, University of São Paulo, São Paulo, Brazil; ^3^Department of Prosthodontics, School of Dentistry, University of São Paulo, São Paulo, Brazil; ^4^Dental Division Research, Guarulhos University, Guarulhos, Brazil

**Keywords:** candidiasis, *Candida albicans*, *Lactobacillus*, immune system, macrophage, pattern recognition receptor

## Abstract

Probiotics are live microorganisms that confer benefits to the host health. The infection rate of potentially pathogenic organisms such as *Candida albicans*, the most common agent associated with mucosal candidiasis, can be reduced by probiotics. However, the mechanisms by which the probiotics interfere with the immune system are largely unknown. We evaluated the effect of probiotic bacteria on *C. albicans* challenged human macrophages. Macrophages were pretreated with lactobacilli alone (*Lactobacillus rhamnosus* LR32, *Lactobacillus casei* L324m, or *Lactobacillus acidophilus* NCFM) or associated with *Escherichia coli* lipopolysaccharide (LPS), followed by the challenge with *C. albicans* or LPS in a co-culture assay. The expression of pattern-recognition receptors genes (*CLE7A, TLR2*, and *TLR4*) was determined by RT-qPCR, and dectin-1 reduced levels were confirmed by flow cytometry. The cytokine profile was determined by ELISA using the macrophage cell supernatant. Overall probiotic lactobacilli down-regulated the transcription of *CLEC7A* (*p* < 0.05), resulting in the decreased expression of dectin-1 on probiotic pretreated macrophages. The tested *Lactobacillus* species down-regulated *TLR4*, and increased *TLR2* mRNA levels in macrophages challenged with *C. albicans*. The cytokines profile of macrophages challenged with *C. albicans* or LPS were altered by the probiotics, which generally led to increased levels of IL-10 and IL-1β, and reduction of IL-12 production by macrophages (*p* < 0.05). Our data suggest that probiotic lactobacilli impair the recognition of PAMPs by macrophages, and alter the production of pro/anti-inflammatory cytokines, thus modulating inflammation.

## Introduction

*Candida albicans* is the most common yeast isolated from the human mucosal surfaces (Scully et al., 1994). Despite its commensal nature, *Candida* causes oral and genital candidiasis that may evolve toward a systemic dissemination. For some decades, antifungals such as nystatin, amphotericin B, and fluconazole have been the drugs of choice for a successful treatment of oral candidiasis (McCullough and Savage, 2005). However, the administration of these agents may result in a temporary gastrointestinal disorder such as nausea, vomiting, and diarrhea (Lewis et al., 1991). The limited spectrum and toxicity of available antifungals, in addition to the gradual emergence of drug-resistant *Candida* strains (Niimi et al., 2010), bring urgency to alternative therapies against fungal infections, such as probiotic bacteria.

Probiotics are defined as live microorganisms that when administered in adequate amounts confer benefits to the host (Sanders and Klaenhammer, 2001). Bacteria belonging to the genus *Lactobacillus* have been used as probiotics due to their association with the healthy gastrointestinal tract in humans (Dunne et al., 1999). Animal (Elahi et al., 2005; Matsubara et al., 2012) and human studies (Hatakka et al., 2007; Ishikawa et al., 2015) have shown that the daily consumption of probiotic bacteria may reduce the oral colonization of *C. albicans*. The available literature supports the use of probiotics in the management of mucosal candidiasis not only in the oral cavity but also in the urogenital system and gastrointestinal tract, both as adjuvant therapy and as a prophylactic agent (Matsubara et al., 2016a). *In vivo* and *in vitro* studies suggest the potential use of probiotics for preventing recurrent vulvovaginal candidiasis (Falagas et al., 2006a), and recurrent urinary tract infections in women (Falagas et al., 2006b). Despite these promising findings, further investigation based on clinical trials is needed to confirm these beneficial effects of probiotics for the human being before its widespread use.

The restoration of a natural healthy microbiome on mucosal surfaces seems to be the major effect of probiotics (Matsubara et al., 2016a). These beneficial microorganisms may control the establishment of opportunistic pathogens, such as *C. albicans*, on mucosal surfaces. This control might be possible by directly interfering with pathogen survival and virulence expression (Ribeiro et al., 2016; Wang et al., 2017), competing for nutrients and adhesion sites or modulating the host immune response (Saarela et al., 2000; Reid et al., 2011; Matsubara et al., 2016b). Probiotic lactobacilli were also found to affect *C. albicans* biofilm formation and filamentation (Matsubara et al., 2016b). The effect of probiotic bacteria can occur by direct interactions between cell surfaces or indirectly through their products (Rizzo et al., 2015), such as lactate, a major fermentation product of lactic acid bacteria (LAB), that regulates critical functions of macrophages and dendritic cells, and also modulates the inflammatory response of epithelial cells (Garrote et al., 2015).

The innate immune system is the first line of defense against pathogens in the mucosa beyond the epithelial barrier (Romani, 2004), and the most important mechanism of protection against disseminated candidiasis (Soloviev et al., 2011). In this scenario, macrophages link the innate and the adaptive immune responses, due to their multifunctional characteristics that include phagocytic and microbicidal activities, a capacity of cytokines production, and antigen presentation (Auwerx, 1991; Romani, 2004).

The capacity of lactobacilli to induce the release of proinflammatory cytokines by macrophages was found to be strain-specific, with lactobacilli cells promoting different responses compared to probiotic cell-free supernatants (Quinteiro-Filho et al., 2015). On the other hand, another study showed that both *Lactobacillus crispatus* cells and its supernatant enhanced the anti-inflammatory response of macrophages and epithelial cell by increasing the production of the regulatory cytokine IL-10 and reducing the proinflammatory cytokines TNF-α and IL-6 (Rizzo et al., 2015).

The recognition of *C. albicans* pathogen-associated molecular patterns (PAMPs) by macrophages is mediated by an array of membrane-bound and soluble pattern-recognition receptors (PRRs) (Brown, 2011), such as Toll-like receptors (TLRs), C-type lectin receptors, including dectin-1, and the galectin family proteins (Taylor et al., 2002). Dectin-1 is the primary receptor for *C. albicans* in phagocytic cells, which recognizes β-glucans of *Candida* cell wall. This recognition leads to an orchestrated signaling that culminates in phagocytosis, antimicrobial effector functions, and production of defensins, chemokines, cytokines and reactive oxygen species (ROS) (Brown, 2011; Romani, 2011). TLR4 in turn not only recognizes mannan of fungi cell wall but also LPS from Gram-negative bacteria (Brown, 2011).

Lactobacilli were found to attenuate the adaptive and innate immune responses in macrophages challenged by LPS or lipoteichoic acid of other bacteria (Pradhan et al., 2016). In turn, specific *Lactobacillus rhamnosus* and *Lactobacillus casei* strains enhanced the phagocytic and microbicidal activities of peritoneal macrophages in mice challenged with *C. albicans* (Marranzino et al., 2012). Despite these evidences, the effects of probiotics on human immune systems affected by *Candida* are is still unclear.

Hence, the present study sought to evaluate the influence of probiotics on the human response to *C. albicans* by determining the effect of three *Lactobacillus* strains on PRR (dectin-1, TLR4, and TLR2) expression, and measuring the impact of these probiotics on inflammatory cytokines production by human macrophages challenged with *C. albicans* and LPS.

## Materials and Methods

### Microorganisms and Culture Conditions

*Candida albicans* ATCC SC5314, isolated from human clinical infection (Jones et al., 2004), and the probiotic strains *L. rhamnosus* LR32, *Lactobacillus acidophilus* NCFM (Danisco, Madison, WI, United States) and *L. casei* L324m (clinical isolate, Institute of Biomedical Sciences, University of São Paulo, Brazil) were used (Matsubara et al., 2016b). All strains were stored in 20% glycerol at -80°C prior to the experiments.

*Candida albicans* was cultivated in Sabouraud dextrose broth (SDB; Difco Laboratories, Detroit, MI, United States) at 37°C, for 18 h in an orbital shaker. Probiotics were cultivated in De Man, Rogosa and Shape broth (MRS; Difco Laboratories) at 37°C, in an anaerobic incubator (85% N_2_, 10% CO_2_, and 5% H_2_) for 18 h.

Probiotic bacteria and yeasts were harvested by centrifugation (2,000 × *g*/5 min), washed twice with phosphate-buffered saline (PBS, pH 7.2), and resuspended at ∼2 × 10^8^ and 6 × 10^6^ CFU/ml, respectively, in RPMI-1640 medium (Sigma–Aldrich, St. Louis, MO, United States) without fetal bovine serum (FBS) and antibiotics.

### Cell Culture

The human macrophage-like cell line THP-1 was grown in RPMI-1640 medium (Sigma–Aldrich, St. Louis, MO, United States) enriched with 10% heat-inactivated FBS (Sigma–Aldrich), 0.05 mM b-mercaptoethanol (Sigma–Aldrich), 11 mM sodium bicarbonate (NaHCO_3_), 1% sodium-pyruvate (Sigma–Aldrich), 10 mM Hepes (Sigma–Aldrich), 1% glutamine (Sigma–Aldrich), and 1% penicillin/streptomycin (Sigma–Aldrich). Cells were incubated at 37°C in a humidified atmosphere containing 10% CO_2_.

### Co-culture Assay

**FIGURE 1:**
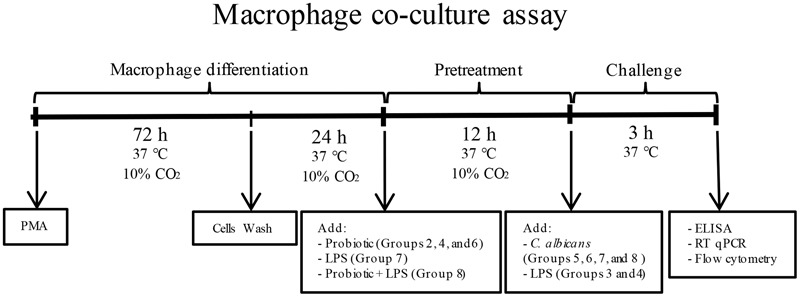
Flowchart of macrophage co-culture assay. Macrophages were pretreated with probiotic lactobacilli alone (Groups 2, 4, and 6), LPS alone (Group 7) or both probiotic and LPS (Group 8). After 12 h of incubation (37°C, 10% CO_2_), macrophages were challenged with *Candida albicans* (Groups 5, 6, 7, and 8) or LPS (Groups 3 and 4). Groups 3 and 5 had no pretreatment, Group 2 received no challenge, and Group 1 was the negative control.

THP-1 cells were plated at 1 × 10^6^ cells/well, in 6-well plates (Corning-Costar, Lowell, MA, United States). The differentiation into macrophages was achieved by treating THP-1 cells with 100 ng/mL phorbol 12-myristate 13-acetate (PMA; Sigma–Aldrich) for 72 h (**Figure 1**). After incubation, plastic-adherent cells were washed twice and culture medium was replaced by RPMI 1640 medium with no FBS and antibiotics, and incubated for 24 h (37°C, 10% CO_2_) (Chanput et al., 2012).

Macrophages were pretreated with probiotic bacteria and/or *Escherichia coli* lipopolysaccharide (LPS) before *C. albicans* or LPS challenge, comprising eight groups of study for each probiotic bacteria as follows: Group 1 – THP-1 (negative control); Group 2 – THP-1 + Probiotic pretreatment; Group 3 – THP-1 + LPS challenge; Group 4 – THP-1 + Probiotic pretreatment + LPS challenge; Group 5 – THP-1 + *Candida* challenge; Group 6 – THP-1 + Probiotic pretreatment + *Candida* challenge; Group 7 – THP-1 + LPS pretreatment + *Candida* challenge; and Group 8 – THP-1 + LPS and Probiotic pretreatments + *Candida* challenge. Macrophages were pretreated with probiotic lactobacilli alone (1 × 10^7^ CFU/well) (Groups 2, 4, and 6), LPS alone (Sigma–Aldrich, 100 ng/ml) (Group 7) or both LPS and probiotic bacteria (Group 8) (Yoon et al., 2013), for 12 h, at 37°C. The cell supernatant of all groups was replaced by fresh medium after pretreatments. Afterward, macrophages were challenged with 50 μl of *C. albicans* suspension (6 × 10^6^ yeast/ml) (Groups 5, 6, 7, and 8) or LPS (100 ng/ml) (Groups 3 and 4) for 3 h, at 37°C. Negative control macrophages received neither pretreatment nor challenge (Group 1). Equivalent total volume was obtained in all groups by the addition of fresh culture medium (RPMI 1640 with no FBS and antibiotics).

Viability of THP-1 cells was maintained after co-culture of macrophages in any of the studied conditions and it was assessed by trypan blue exclusion assay (Barker et al., 2005) (**Supplementary Figure S1**). Relative transcription of *CLEC7A, TLR4* and *TLR2*, expression of dectin-1 on macrophage surface, and cytokines production were evaluated after the final incubation. All assays were carried out in triplicate in three independent assays.

### Relative Transcription of *CLEC7A, TLR4*, and *TLR2*

Macrophages were lysed and total RNA was extracted using RNeasy KIT (QIAGEN, Valencia, CA, United States). The concentration, purity, and quality of the isolated RNA samples were determined using a Nano DropOne/One Spectrophotometer (Thermo Scientific, Waltham, MA, United States). The RNA from each sample was immediately reverse transcribed into cDNA using SuperScript VILO MasterMix (Invitrogen, Waltham, MA, United States). The conditions for reverse transcription were 15 min at 37°C, 5 s at 85°C. Relative expression levels of *CLEC7A, TLR4*, and *TLR2* were evaluated by quantitative real-time reverse transcription PCR (RT-qPCR), using TaqMan Gene Expression Master Mix (Applied Biosciences, Foster City, CA, United States), TaqMan primers and probes for *CLEC7A* (*Hs01902549_sl*), *TLR4* (*Hs01060206_m1*), *TLR2* (*Hs00152932_m1*), and *GAPDH* (*Hs02758991_gl*) and 100 ng of cDNA in each reaction. The RT-qPCR comprised an initial step of 50°C for 2 min, 95°C for 10 min followed by 40 cycles at 95°C for 15 s, and 50°C for 1 min, using Step One Plus System (Applied Biosciences). All data were normalized to *GAPDH* transcripts levels in the same cDNA set (Pfaffl, 2001; Gantner et al., 2005).

### Expression of Dectin-1 on Macrophages Surface

After the final incubation, macrophages were washed with PBS and treated with Accutase^TM^ cell detachment solution (BD Biosciences, San Jose, CA, United States). Cells were incubated with Fc Block^TM^ (Human BD Fc Block, BD Biosciences) and stained with either an anti-human Clec7a – Dectin-1 antibody or an isotype control (5 μl/million cells, both labeled with PE from BD Biosciences) (30 min, 4°C in the dark) (**Supplementary Figure S2**). After washing with PBS, stained cells were analyzed by fluorescence-activated cell sorting (FACS) for at least 10,000 events (Guava easyCyte^TM^ Flow Cytometers – Merck Millipore, Merck KgaA, Darmstadt, Germany).

### Cytokine Profile

The concentrations of TNF-α, IL-10, IL-12 (p70), and IL-1β secreted by macrophages after 3 h of co-culture were determined in macrophage cells supernatants by ELISA (BD Biosciences, San Jose, CA, United States) following the manufacturer’s recommendation. The cytokines levels were determined by comparison with a standard calibration curve.

### Statistical Analysis

Data were expressed as mean ± standard deviation (SD) from three independent experiments. One-way ANOVA test followed by Tukey’s Multiple Comparison test was used for statistical analyses. Statistical significance was set at *p* < 0.05 (GraphPad Prism^®^ Version 6.0c – GraphPad Software, La Jolla, CA, United States).

## Results

### Probiotics Alter the Cytokine Profile of Macrophages Challenged with LPS and/or *Candida albicans*

**FIGURE 2:**
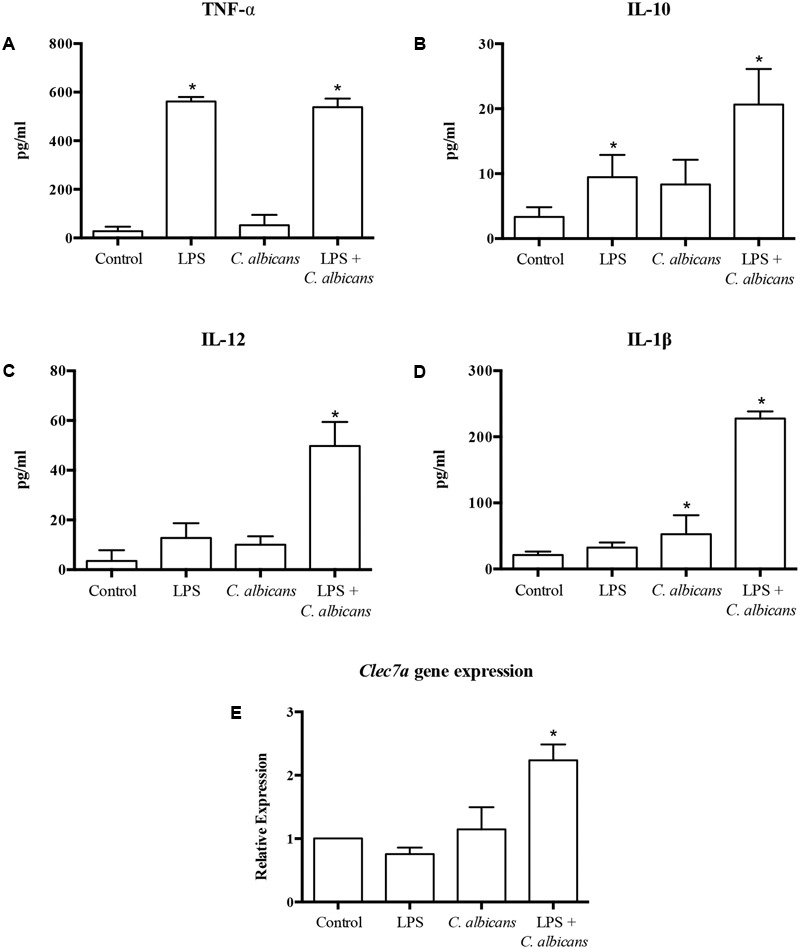
Cytokine production [TNF-α **(A)**, IL-10 **(B)**, IL-12 **(C)**, and IL-1β **(D)**], and transcription of *CLEC7A* **(E)** by macrophages challenged with LPS, *C. albicans*, or pretreated with LPS and challenged by *C. albicans* (LPS + *C. albicans*). Control group had neither challenge nor pretreatment. Data are presented as mean ± SD of three independent experiments. ^∗^*p* < 0.05.

In order to evaluate the effect of probiotic bacteria on macrophages challenged with *C. albicans* and/or LPS, we first investigated the impact of *C. albicans* and LPS challenges on cytokine production by macrophages (**Figure 2**). The challenge with *C. albicans* alone was not able to induce a significant production of TNF-α, IL-10, and IL-12 (*p* > 0.01) as compared to control non-challenged macrophages, although the stimulus with *C. albicans* increased the level of IL-1β significantly (*p* < 0.01). In contrast, TNF-α and IL-10 levels were dramatically increased (*p* > 0.01) in the supernatants of macrophages challenged with LPS, but the same effect was not observed for IL-12 and IL-1β levels. The pretreatment with LPS before the challenge with *C. albicans* promoted a synergistic effect on macrophages when compared to macrophages challenged with *C. albicans* or LPS alone (except for TNF-α), inducing the highest levels of proinflammatory cytokines among the given groups. This was associated with an increase in the expression of *clec7a* (**Figure 2E**) in macrophages pretreated with LPS before *C. albicans* challenge.

**FIGURE 3:**
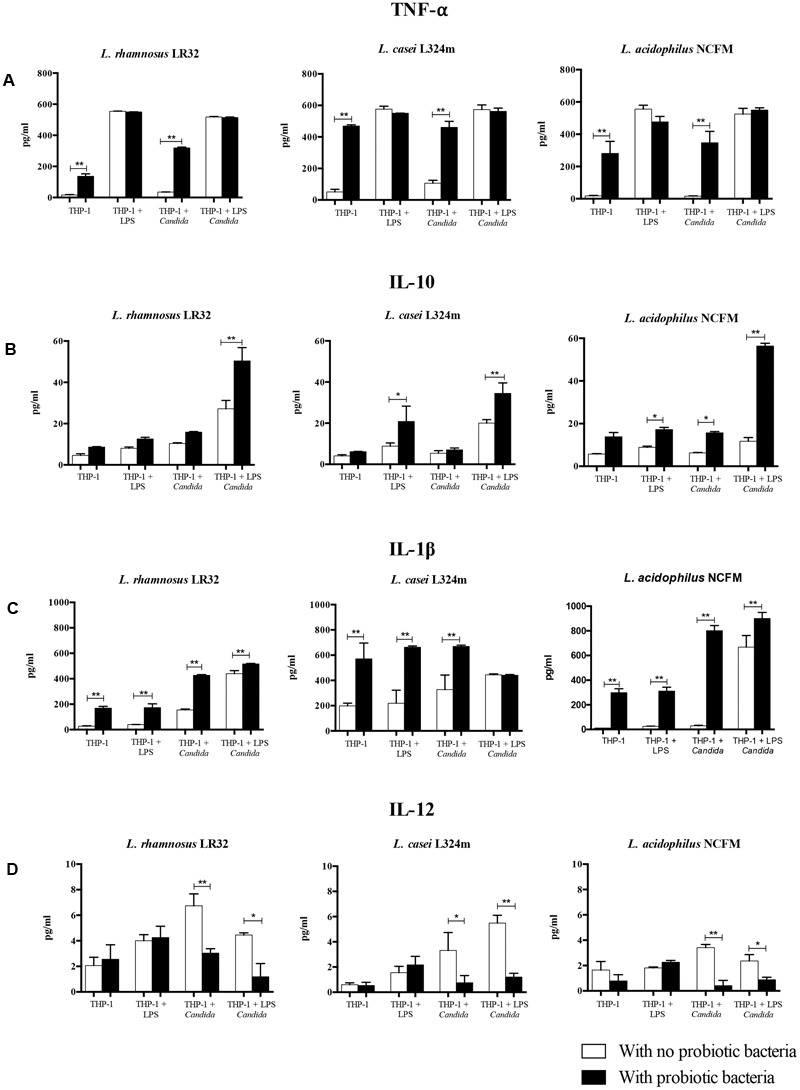
Effect of probiotic bacteria pretreatment on the production of TNF-α **(A)**, IL-10 **(B)**, IL-1β **(C)**, and IL-12 **(D)** by macrophages. The quantification of cytokines in the macrophage supernatants was determined by ELISA. Eight groups of study were performed for each *Lactobacillus* strain tested: Group 1 – THP-1 (negative control); Group 2 – THP-1 + Probiotic pretreatment; Group 3 – THP-1 + LPS challenge; Group 4 – THP-1 + Probiotic pretreatment + LPS challenge; Group 5 – THP-1 + *Candida* challenge; Group 6 – THP-1 + Probiotic pretreatment + *Candida* challenge; Group 7 – THP-1 + LPS pretreatment + *Candida* challenge; and Group 8 – THP-1 + LPS and Probiotic pretreatments + *Candida* challenge. Three different strains of probiotic bacteria were tested: *Lactobacillus rhamnosus* LR32, *Lactobacillus casei* L324m, and *Lactobacillus acidophilus* NCFM. Data are presented as mean ± SD of three independent experiments. ^∗^*p* < 0.05; ^∗∗^*p* < 0.01.

The treatment of macrophages with probiotics affected the production of cytokines significantly, with similar performances for all three tested probiotic strains (**Figure 3**). The pretreatment with probiotic lactobacilli (Group 2) induced a significant production of TNF-α and IL1-β as compared to non-treated macrophages (Group 1). The pretreatment with probiotics on LPS challenged macrophages caused a substantial increase in the levels of IL1-β and IL-10, except for *L. rhamnosus* pretreated macrophages. On the contrary, the production of TNF-α and IL-12 by LPS stimulated macrophages was not affected by probiotic bacteria.

In turn, the probiotic pretreatment of macrophages challenged with *C. albicans* (Group 6) led to increased levels of TNF-α and IL-1β (*p* < 0.01), while the secretion of IL-10 was increased significantly only by *L. acidophilus* pretreated macrophages. In contrast, *L. rhamnosus, L. acidophilus*, and *L. casei* pretreatments reduced significantly (*p* < 0.05) the levels of IL-12. In the most complex situation simulated in our study, the effects of probiotics on increasing the production of IL-10 and IL-1β (except for *L. casei*), and decreasing IL-12 were observed in cells pretreated with LPS and challenged by *C. albicans*, in different levels according to the probiotic strain.

### Probiotics Reduce Transcription of *CLEC7A* and Dectin-1 Expression in Macrophages

**FIGURE 4:**
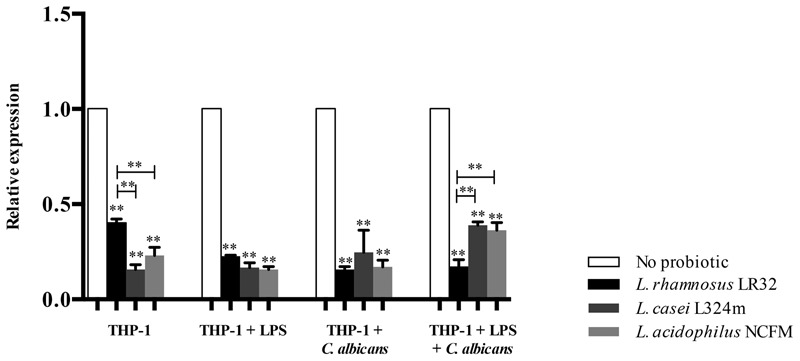
Relative transcription of *CLEC7A* in macrophages pretreated with probiotics alone or with both LPS and probiotics for 12 h before being challenged with *C. albicans* or LPS for 3 h. *L. rhamnosus* LR32, *L. casei* L324m, and *L. acidophilus* NCFM were tested in these different situations. The transcription of *CLEC7A* was normalized to *GAPDH* reference gene (internal control). Groups with no probiotic bacteria pretreatment were set at 1. Data are presented as mean ± SD. ^∗∗^*p* < 0.01.

The pretreatment of macrophages with all three tested probiotics down-regulated the transcription of *CLEC7A* (*p* < 0.01) (**Figure 4**), which codifies dectin-1 receptors. *L. rhamnosus* was the most effective in down-regulating *CLEC7A* (*p* < 0.01) in macrophages pretreated with LPS and challenged with *C. albicans* compared to the remaining *Lactobacillus* strains. However, both *L. acidophilus* and *L. casei* induced higher down-regulation of *CLEC7A* than *L. rhamnosus* in macrophages with no additional challenge (**Figure 4**).

**FIGURE 5:**
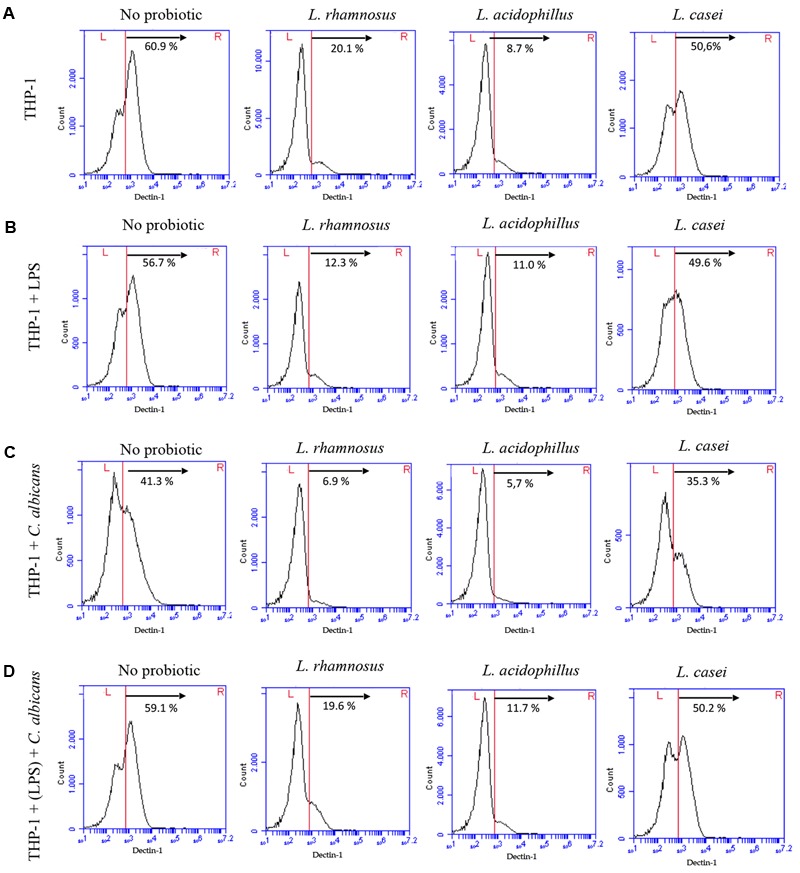
Expression of dectin-1 receptors by THP-1 macrophages in control groups (no probiotic pretreatment) and test groups (pretreated with *L. rhamnosus* LR32, *L. acidophilus* NCFM, or *L. casei* L324m) determined by flow cytometry. **(A)** Macrophages pretreated with probiotic bacteria; **(B)** macrophages pretreated with probiotic bacteria and challenged with LPS; **(C)** macrophages pretreated with probiotic and challenged with *C. albicans*; **(D)** macrophages pretreated with both LPS and probiotic before *C. albicans* challenge. Percentage of cells expressing high levels of dectin-1 is shown for each situation.

The reduced expression of dectin-1 receptor on macrophage surfaces promoted by probiotics was confirmed by flow cytometry (**Figure 5**). *L. rhamnosus* and *L. acidophilus* presented similar percentage of cells expressing dectin-1, whereas *L. casei* was found to interfere to a lesser extent under all simulated conditions. The used antibody had no non-specific binding to either *C. albicans* or *Lactobacillus* cells (**Supplementary Figure S3**).

### Probiotics Down-Regulate *TLR4* and Up-Regulate *TLR2* in Macrophages Challenged with *C. albicans*

**FIGURE 6:**
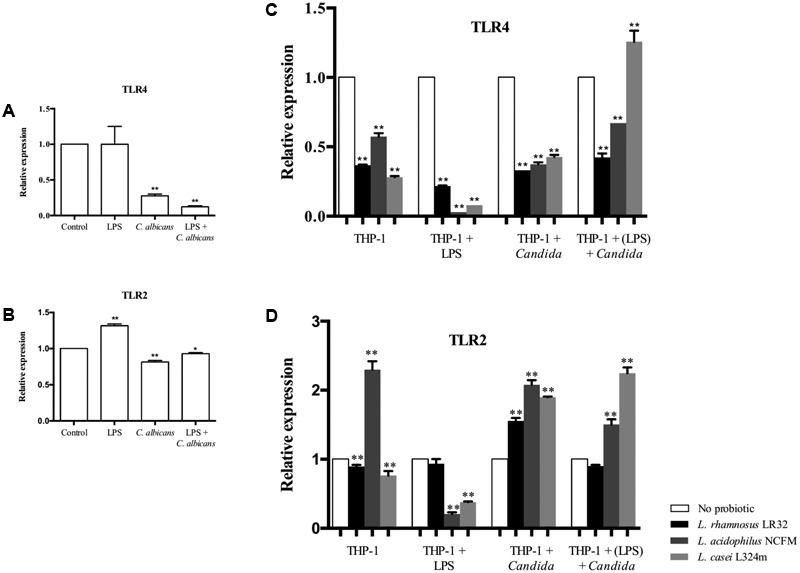
Relative transcription of *TLR4* **(A)** and *TLR2* **(B)** in macrophages challenged with LPS, *C. albicans*, or pretreated with LPS before *C. albicans* challenge. Transcription of *TLR4* **(C)** and *TLR2* **(D)** in macrophages pretreated with probiotics alone or both LPS and probiotics before *C. albicans* or LPS challenge. Gene expression was normalized to *GAPDH* transcripts levels in the same cDNA set. Groups with no probiotic bacteria pretreatment were set at 1. Data are presented as mean ± SD. ^∗^*p* < 0.05; ^∗∗^*p* < 0.01.

The challenge of macrophages with *C. albicans* was found to down-regulate *TLR4* (**Figure 6A**). The tested *Lactobacillus* strains further reduced the transcription of this gene in macrophages stimulated by *C. albicans*, except for *L. casei* that increased *TLR4* mRNA levels when the macrophages were pretreated with LPS and challenged by *C. albicans* (**Figure 6C**). In turn, the LPS challenge alone was not able to alter the transcription of *TLR4* after 3 h of incubation (**Figure 6A**), but a significant decrease in the transcription of *TLR4* was observed when these macrophages were pretreated with probiotics (**Figure 6C**).

Macrophages challenged with LPS alone presented up-regulation of *TLR2*, while a reduced expression of this gene was observed when macrophages were challenged with *C. albicans* (**Figure 6B**). However, the expression of *TLR2* was entirely changed by probiotic pretreatments as the transcription of *TLR2* was up-regulated in *C. albicans* challenged macrophages for all three *Lactobacillus* strains tested (**Figure 6B**). Probiotics also promoted a significant (*p* < 0.01) decrease in *TLR2* mRNA levels when the macrophages were challenged with LPS, except for macrophages pretreated with *L. rhamnosus*. It is noteworthy that the pretreatment of macrophages with *L. acidophilus* alone showed a unique effect increasing *TLR2*, while the other probiotic bacteria (*L. rhamnosus* and *L. casei*) reduced the transcription of this gene.

## Discussion

The reduction of *C. albicans* oral colonization promoted by LAB, such as *Lactobacillus* species, has already been demonstrated in animal models (Matsubara et al., 2012) and clinical trials (Ishikawa et al., 2015). However, the mechanisms underlying this beneficial effect are still poorly understood, especially from the human immune system perspective. In the present study, we investigated how *Lactobacillus* species influence the immune response of human macrophages challenged by *C. albicans*, analyzing two important factors for the macrophages activity: production of pro- and anti-inflammatory cytokines and expression of receptors for *Candida* recognition.

Our study was designed to simulate different conditions encountered in the human mucosa. The LPS, during both pretreatment or challenge stimulus, represented Gram-negative bacteria colonizing the mucosal surfaces. The pretreatment of macrophages with LPS aimed to enhance the response of macrophages to *C. albicans* as demonstrated previously (Rogers et al., 2013). The LPS challenge, in turn, acted as a positive control to the *C. albicans* challenge groups. Groups 7 and 8 simulated the clinical situation where macrophages are continuously stimulated by endogenous bacteria before the overgrowth of *C. albicans*.

Macrophages with no probiotic pretreatment (Groups 1, 3, 5, and 7), challenged with *C. albicans* alone, produced only significant levels of IL-1β (**Figure 2D**). However, the pretreatment of macrophages with LPS enhanced the response to *C. albicans*, which not only expanded the production of TNF-α, IL-1β and IL-12, but also increased the secretion of the regulatory cytokine IL-10 (**Figures 2A–D**). It has already been demonstrated that the presence of bacterial LPS induces the expression of dectin-1 on macrophages, which in turn leads to an enhanced immune response against *C. albicans* (Rogers et al., 2013). This evidence corroborates with our results of up-regulation of transcription of *CLEC7A* in LPS challenged macrophages (**Figure 2E**).

In lactobacilli pretreated macrophages, the probiotics supplied an additional ‘reprogramming’ stimulus suggesting their immunomodulatory effect, due to decreased production of IL-12, and increased levels of IL-10. The interference with the release of pro- and anti-inflammatory cytokines by human macrophages is probiotic-strain dependent, since strains of the same species may show opposite effects (Drago et al., 2010). Other lactobacilli species, such as *L. crispatus*, an important urogenital species routinely found in the vagina of healthy women, also enhanced the production of the IL-10, and reduced the proinflammatory cytokine production in macrophages and epithelial cells challenged with *Chlamydia trachomatis* (Rizzo et al., 2015). This immunomodulatory effect induced by probiotic bacteria may lead to an enhanced capacity of macrophages to combat *C. albicans* infections. A previous *in vivo* study using a *C. albicans* infection murine model showed significant increase in the phagocytic and microbicidal activities of peritoneal macrophages of mice treated with *L. rhamnosus* and *L. casei*, which resulted in decreased levels of the pathogen in infected organs (Marranzino et al., 2012).

IL-10 is one of the most important immunosuppressive cytokines, aiming the maintenance of homeostasis (Ma et al., 2015), and its production can be enhanced by probiotics (Lucas et al., 2005). In turn, during pathogen infections, IL-12 produced by macrophages/monocytes and dendritic cells (Trinchieri, 1995) influences the production of interferon-gamma (IFN-γ) by natural killer (NK) and T cells, which favor phagocytic cells activation and inflammation. IL-12 also plays an important role by favoring a T helper type 1 (Th1) response (Trinchieri, 1993). In our study, the reduction of IL-12 promoted by probiotics may be also associated with the increase in IL-10 levels, since IL-10 is a potent inhibitor of IL-12 production by human peripheral blood mononuclear cells (D’Andrea et al., 1993), by suppressing IL-12 production at the transcriptional level (Aste-Amezaga et al., 1998).

The significant increase of IL1-β production by lactobacilli-treated macrophages after *C. albicans* challenge (**Figure 3C**) may suggest that probiotic bacteria are activating inflammasome, as shown for *L. rhamnosus* (Miettinen et al., 2012). Since inflammasomes contribute to an anti-*Candida* response (Martinon et al., 2002), probiotic treatment may increase *Candida* elimination.

In our co-culture assay, *L. rhamnosus* LR32, *L. casei* L324m, and *L. acidophilus* NCFM induced similar cytokine profile in human macrophages, although the level of inhibition (e.g., IL-12) or stimulation (e.g., IL-10) in cytokines production varied according to the tested bacterial strain. These differences may be associated with their mechanisms to modulate TLR expression in phagocytic cells (Kotzamanidis et al., 2010). In fact, the tested *L. rhamnosus* strain down-regulated the expression of *TLR4, L. casei* up-regulated *TLR4* and *TLR2*, whereas *L. acidophilus* decreased *TLR4* and increased *TLR2* transcription levels in *C*. *albicans* challenged macrophages.

The decreased expression of *TLR4* in LAB pretreated macrophages observed in our study is in accordance with previous *in vivo* data reporting that probiotic lactobacilli reduce *TLR4* mRNA levels and decrease TLR2 responses in blood mononuclear cells (Forsberg et al., 2014). In another study, the pretreatment with *L. crispatus* also downregulated the expression of TLR4 and TLR2 by epithelial cells challenged with *C. albicans*, suggesting that *L. crispatus* may act as an anti-inflammatory agent through TLR2/4 (Rizzo et al., 2013). Since TLR4 signaling culminates in the expression of genes encoding inflammatory molecules (Aksoy et al., 2012), the down-regulation of *TLR4* transcription by *L. rhamnosus* and *L. acidophilus* favors an anti-inflammatory effect, reducing the production of proinflammatory cytokines such IL-12 (Re and Strominger, 2001).

Our data also indicated that probiotics increased *TLR2* mRNA levels in *C. albicans* challenged macrophages (**Figure 6D**). *C. albicans* cell wall mannan and phospholipomannan are able to trigger TLR2-signaling (Brown, 2011), but the role of TLR2 signaling in controlling *C. albicans* infections is still contradictory. Increased susceptibility or resistance to infection with *C. albicans* were both showed in TLR2-deficient mice, due to reduced proinflammatory cytokines production or decreased IL-10/increased IL-12 and IFN-γ production, respectively (Brown, 2011). TLR2 and TLR4 signaling are not equivalent as TLR2 stimulus elicits less IL-12 (p70) than TLR4 (Re and Strominger, 2001), but induces abundant IL-10, favoring Th2 and T cytotoxic responses (Dillon et al., 2004). Thus, the up-regulation of *TLR2* by *Lactobacillus* spp. in the presence of *C. albicans* (**Figure 6D**) may partially explain the cytokine profile observed in our study (**Figures 3B,D**), with high IL-10 and low IL-12 levels after probiotics treatment.

All three tested probiotics down-regulated dectin-1 expression in *Candida* challenged macrophages. Even though bacterial LPS induces dectin-1 expression in macrophages (Rogers et al., 2013), the probiotic pretreatments were able to reduce dectin-1 expression even in LPS challenged macrophages (**Figure 4**), by down-regulating the transcription of *CLEC7A*. The importance of dectin-1 signaling to control commensal *C. albicans* levels in the gastrointestinal tract is still controversial. Individuals with genetic deficiencies associated with decreased expression of dectin-1 are more susceptible to fungal diseases (Romani, 2011). On the other hand, in an animal model, dectin-1 was found to be essential for controlling infection of the gastrointestinal tissues during systemic candidiasis, but it had no influence on gastrointestinal colonization of *C. albicans* in mice (Vautier et al., 2012).

The decrease of dectin-1 expression in macrophages promoted by *L. rhamnosus* and *L. acidophilus*, and to a lesser extent by *L. casei*, showed in our study, may indicate that these beneficial bacteria may limit an exacerbated inflammatory response originated from the recognition of *C. albicans* yeasts by macrophages. This inflammatory control may avoid tissue destruction that facilitates the establishment of *C. albicans* infections, but still allowing host defense mechanisms.

This hypothesis is reinforced by a previous study showing that the dectin-1 level reduction is associated with an increased regulatory T cells differentiation and a local homeostasis, thus leading to ameliorate colitis (Tang et al., 2015). The down-regulation of pro-inflammatory genes was also observed in murine macrophages treated with *L. acidophilus* (Pradhan et al., 2016). Furthermore, the capacity of probiotics to reduce yeast-to-hyphae transition of *C. albicans*, as demonstrated previously by our group (Matsubara et al., 2016b), may also contribute to the reduction of local inflammation, as *Candida* remains in the commensal yeast form.

The alteration of *C. albicans* recognition by immune cells in the presence of lactobacilli may involve other mechanisms. For instance, the exposure of *C. albicans* to lactate, produced by LAB, induces β-glucan masking in *C. albicans* by changing the expression of cell-wall-related genes (Ballou et al., 2016). As the fungal cell wall architecture play an essential role in the competitive colonization of *Candida* species in mucosal surfaces (Sem et al., 2016), the effect of probiotic bacteria on fungal cell wall needs to be further investigated.

## Conclusion

**FIGURE 7:**
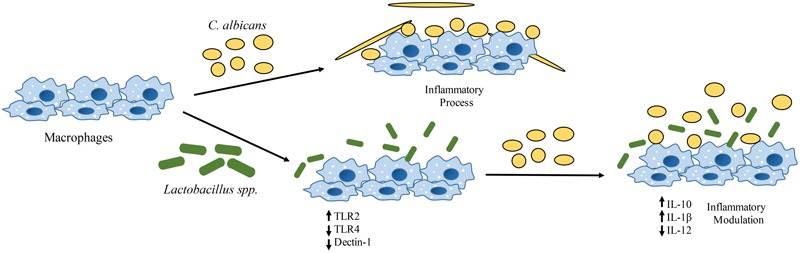
Effects of *Lactobacillus* spp. on human macrophages challenged with *C. albicans*. There is an alteration in the transcription of pattern-recognition receptors (PRRs) genes (*CLEC7A, TLR2*, and *TLR4*) with a significant reduction of *C. albicans* receptors (dectin-1) on macrophages. These changes have an impact on the cytokine profile that leads to an anti-inflammatory effect.

Lactobacilli may contribute in the response against *C. albicans* colonization and infection by affecting the expression of dectin-1, TLR2, and TLR4 at the transcription level, thus altering the recognition of *C. albicans* and the cytokine profile of macrophages. This immunomodulation favors an anti-inflammatory response to *C. albicans* colonization (**Figure 7**). Our novel findings open new possibilities for the study of probiotics against *C. albicans* infections. Further investigations are necessary to determine whether the immune modulation is caused by a direct or an indirect effect of probiotic bacteria on macrophages. More studies testing other immune cells and analyzing different inflammatory signaling pathways will help to clarify the role of probiotic bacteria on *Candida*-host interactions.

## Ethics Statement

This article does not contain any studies with human participants or animals performed by any of the authors.

## Author Contributions

VM designed and conducted experiments, analyzed data and wrote the article; KI helped with flow cytometer assay; EA-S helped with RT-qPCR assay; BB-S helped with cell culture experiments; AN gave a critical review of the manuscript; MM supervised the project and the article writing.

## Conflict of Interest Statement

The authors declare that the research was conducted in the absence of any commercial or financial relationships that could be construed as a potential conflict of interest.

## Supplementary Material

FIGURE S1 Number of viable macrophage cells after the final incubation in the different groups. Data are presented as cells/well.

FIGURE S2 **(A)** Macrophages under different stimulation (cell with no challenge or LPS pretreated cells challenged with *C. albicans*) incubated with isotype control antibody. **(B)** Flow cytometry gating on macrophages.

FIGURE S3 Flow cytometry data from control wells containing *C. albicans* or *Lactobacillus* strains submitted to anti-Human CLEC7A – Dectin-1 antibody. **(A)** macrophages alone with no antibody; **(B)** *C. albicans* + antibody; **(C)** *L. rhamnosus* + antibody; **(D)** *L. acidophilus* + antibody; **(E)** *L. casei* + antibody. Percentage of cells expressing the antibody, shown in each situation, reveals no non-specific binding in any control group.
