# Supplementary figures and images for: Probiotic Bacteria Alter Pattern-Recognition Receptor Expression and Cytokine Profile in a Human Macrophage Model Challenged with Candida albicans and Lipopolysaccharide
Source: Front Microbiol. 2017 Nov 29;8:2280. doi: 10.3389/fmicb.2017.02280 (PMC5712552; doi:10.3389/fmicb.2017.02280)

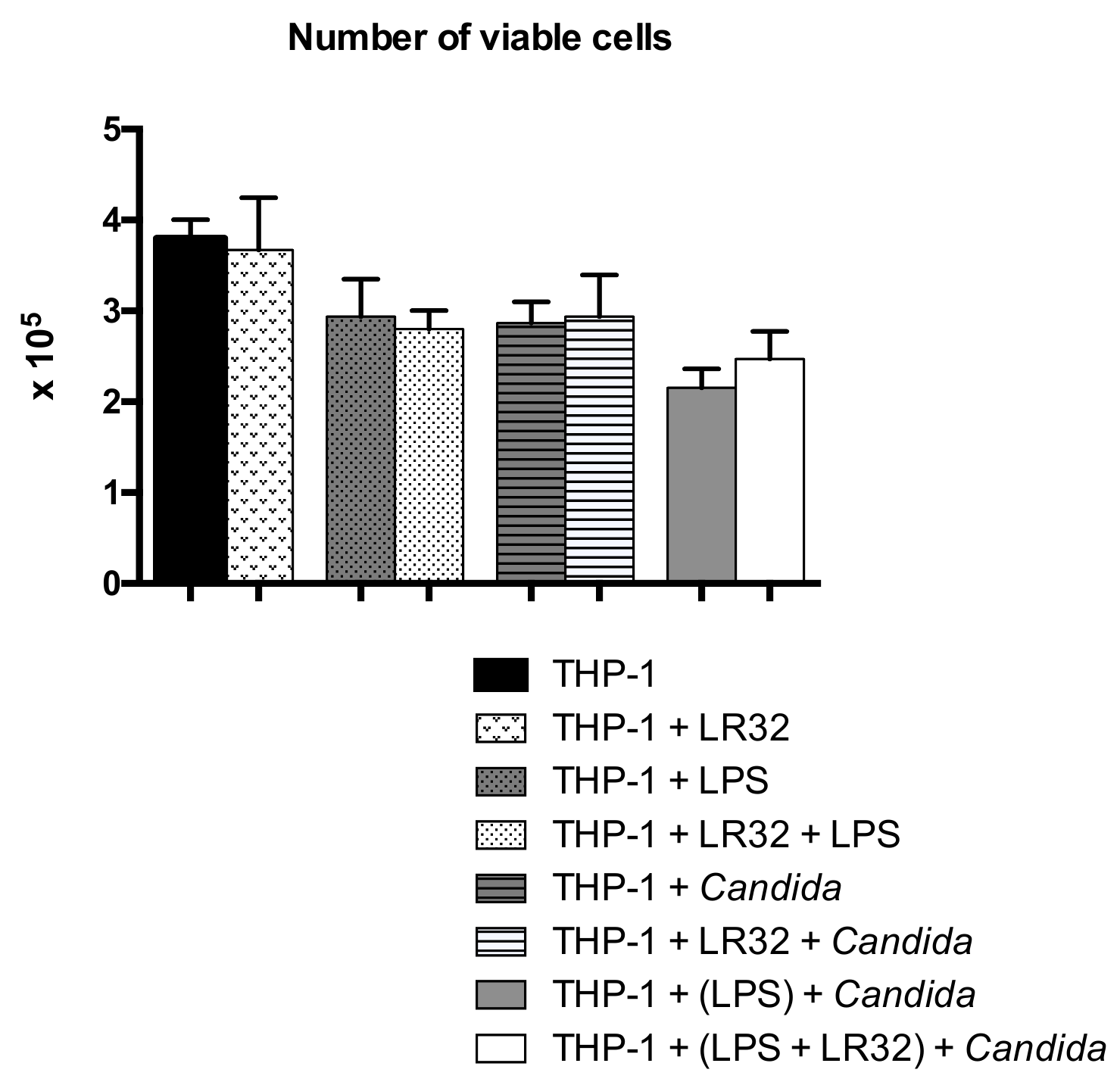

Supplement: FIGURE S1 — Number of viable macrophage cells after the final incubation in the different groups. Data are presented as cells/well. [file Image_1.tiff]

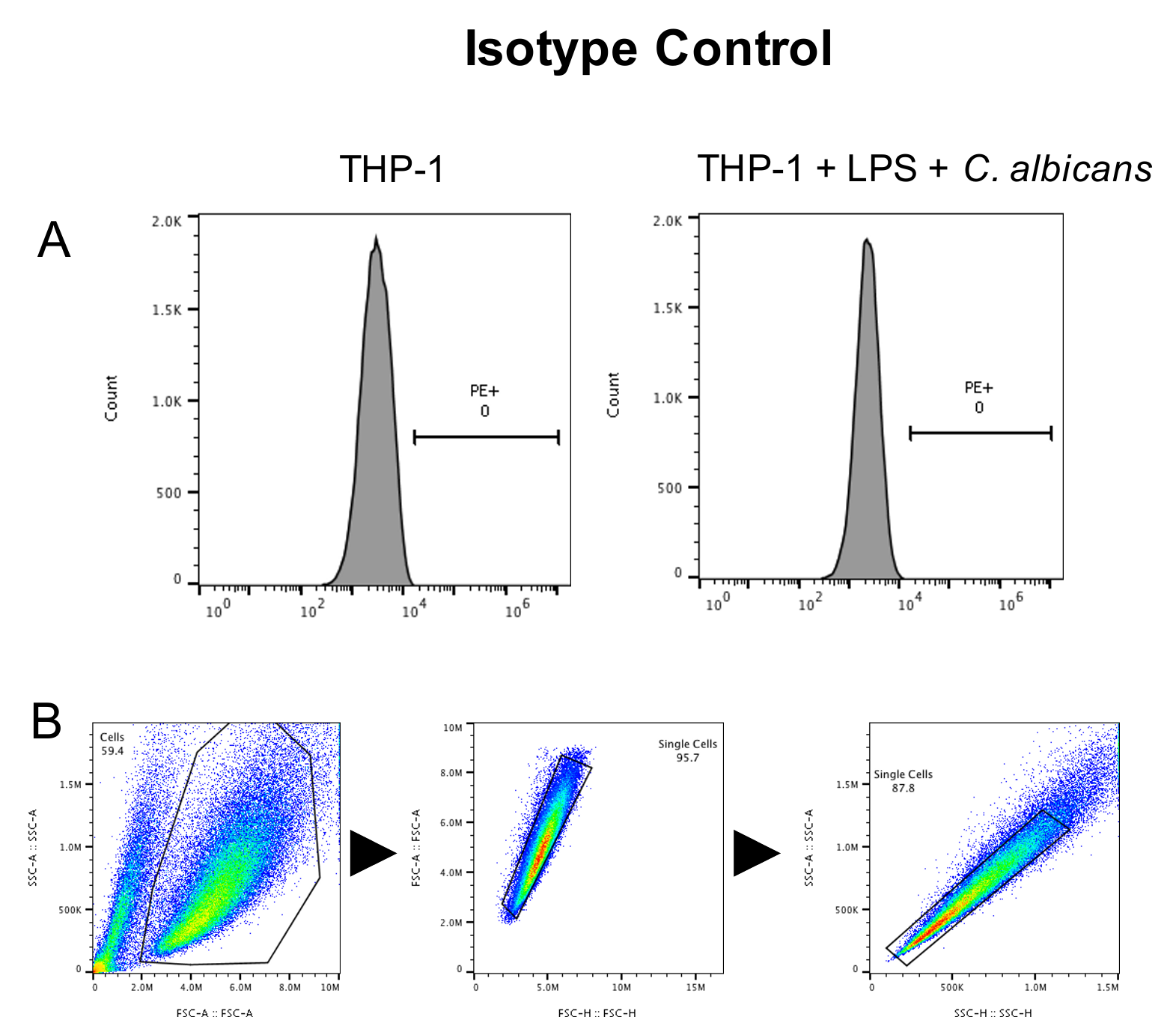

Supplement: FIGURE S2 — (A) Macrophages under different stimulation (cell with no challenge or LPS pretreated cells challenged with C. albicans) incubated with isotype control antibody. (B) Flow cytometry gating on macrophages. [file Image_2.tiff]

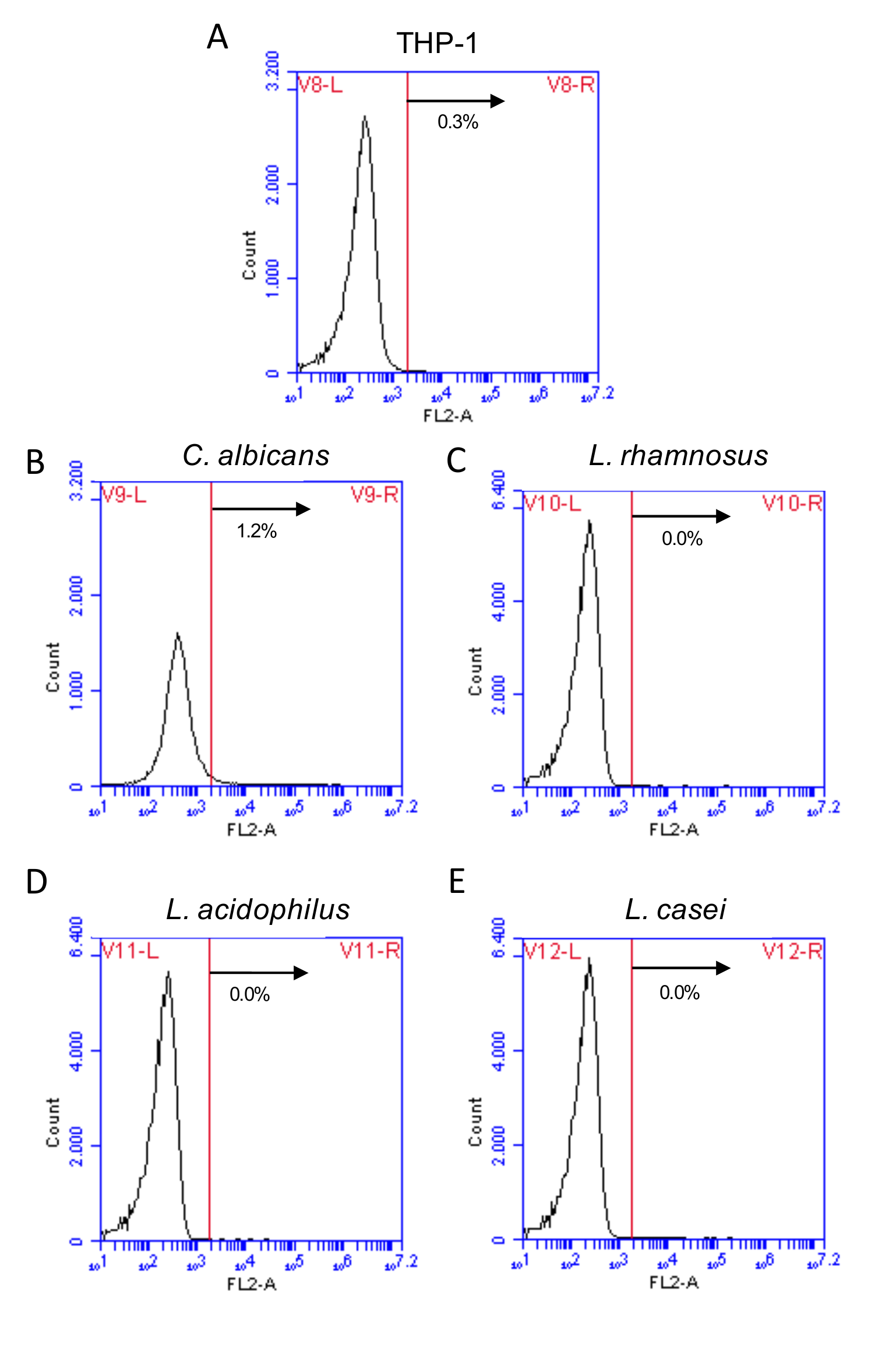

Supplement: FIGURE S3 — Flow cytometry data from control wells containing C. albicans or Lactobacillus strains submitted to anti-Human CLEC7A – Dectin-1 antibody. (A) macrophages alone with no antibody; (B) C. albicans + antibody; (C) L. rhamnosus + antibody; (D) L. acidophilus + antibody; (E) L. casei + antibody. Percentage of cells expressing the antibody, shown in each situation, reveals no non-specific binding in any control group. [file Image_3.tiff]
